# Metabolism of 2,3-Dehydrosilybin A and 2,3-Dehydrosilybin B: A Study with Human Hepatocytes and Recombinant UDP-Glucuronosyltransferases and Sulfotransferases

**DOI:** 10.3390/antiox10060954

**Published:** 2021-06-14

**Authors:** Jiří Vrba, Barbora Papoušková, Kateřina Lněničková, Pavel Kosina, Vladimír Křen, Jitka Ulrichová

**Affiliations:** 1 Department of Medical Chemistry and Biochemistry, Faculty of Medicine and Dentistry, Palacký University, Hněvotínská 3, 77515 Olomouc, Czech Republic; katerina.lnenickova@upol.cz (K.L.); pavel.kosina@upol.cz (P.K.); jitka.ulrichova@upol.cz (J.U.); 2 Department of Analytical Chemistry, Faculty of Science, Palacký University, 17. Listopadu 12, 77146 Olomouc, Czech Republic; barbora.papouskova@upol.cz; 3 Laboratory of Biotransformation, Institute of Microbiology of the Czech Academy of Sciences, Vídeňská 1083, 14220 Prague, Czech Republic; kren@biomed.cas.cz

**Keywords:** dehydrosilybin, silybin, metabolism, glucuronidation, sulfation, UDP-glucuronosyltransferase, sulfotransferase

## Abstract

2,3-Dehydrosilybin A and 2,3-dehydrosilybin B are a pair of enantiomers formed by the oxidation of the natural flavonolignans silybin A and silybin B, respectively. However, the antioxidant activity of 2,3-dehydrosilybin molecules is much stronger than that of their precursors. Here, we investigated the biotransformation of pure 2,3-dehydrosilybin A and 2,3-dehydrosilybin B in isolated human hepatocytes, and we also aimed to identify human UDP-glucuronosyltransferases (UGTs) and sulfotransferases (SULTs) with activity toward their respective enantiomers. After incubation with hepatocytes, both 2,3-dehydrosilybin A and 2,3-dehydrosilybin B were converted to hydroxyl derivatives, methylated hydroxyl derivatives, methyl derivatives, sulfates, and glucuronides. The products of direct conjugations predominated over those of oxidative metabolism, and glucuronides were the most abundant metabolites. Furthermore, we found that recombinant human UGTs 1A1, 1A3, 1A7, 1A8, 1A9, and 1A10 were capable of catalyzing the glucuronidation of both 2,3-dehydrosilybin A and 2,3-dehydrosilybin B. UGTs 1A1 and 1A7 showed the highest activity toward 2,3-dehydrosilybin A, and UGT1A9 showed the highest activity toward 2,3-dehydrosilybin B. The sulfation of 2,3-dehydrosilybin A and B was catalyzed by SULTs 1A1*1, 1A1*2, 1A2, 1A3, 1B1, 1C2, 1C4, and 1E1, of which SULT1A3 exhibited the highest activity toward both enantiomers. We conclude that 2,3-dehydrosilybin A and B are preferentially metabolized by conjugation reactions, and that several human UGT and SULT enzymes may play a role in these conjugations.

## 1. Introduction

**Figure 1:**
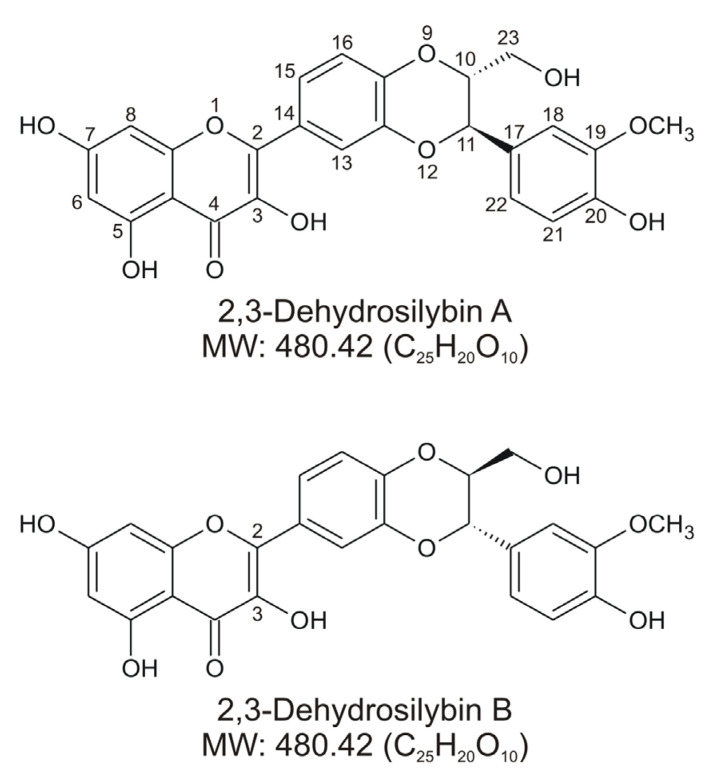
Chemical structures of 2,3-dehydrosilybin A and 2,3-dehydrosilybin B.

2,3-Dehydrosilybin is a mixture of two enantiomers, designated 2,3-dehydrosilybin A and 2,3-dehydrosilybin B (Figure 1), derived from the oxidation of the natural flavonolignans silybin A and silybin B, respectively. Silybin A and B, which are biosynthetically derived from the flavonoid taxifolin and coniferyl alcohol, are among the main bioactive components of hepatoprotective preparations containing silymarin, an extract from the fruits of the plant *Silybum marianum* (milk thistle) [1]. Although 2,3-dehydrosilybin A and B are mostly found as minor constituents in silymarin, it is still unclear whether they occur naturally in living plants or whether they are formed only during processing and storage of the plant material and related products [2,3]. The flavonoid moiety in the molecules of 2,3-dehydrosilybin is formally derived from quercetin, and it is not surprising that research has initially focused on its antioxidant activity. Under in vitro conditions, 2,3-dehydrosilybin showed higher radical scavenging and anti-lipid peroxidation effects than silybin [4,5]. It was also found that 2,3-dehydrosilybin attenuated cell damage associated with oxidative stress induced by various stimuli, including prooxidant substances [4,6], hypoxia/reoxygenation [7], and UV light irradiation [8]. Moreover, hepatoprotective [6,9,10], cardioprotective [11], and potentially anticancer effects [12,13,14] have been reported for 2,3-dehydrosilybin in various experimental models. This bioactive potential also stimulated research focused on the preparation and biological evaluation of some derivatives of 2,3-dehydrosilybin, such as galloyl esters [13] and alkyl ethers [15].

In recent years, research interest in 2,3-dehydrosilybin has naturally focused on its metabolism. It has been shown in vitro that the biotransformation of 2,3-dehydrosilybin may involve cytochrome P450 (CYP) enzymes responsible for the oxidative (phase I) metabolism of drugs and other xenobiotics. Human recombinant CYPs 1A2, 2A6, 2B6, 2C8, 2C9, 2C19, 2D6, 2E1, and 3A4 have been found to hydroxylate 2,3-dehydrosilybin, and the latter has also been shown to catalyze its *O*-demethylation [16]. Demethylation was also found to occur during the anaerobic conversion of 2,3-dehydrosilybin by the human fecal microbiota [17]. On the other hand, experiments with isolated human hepatocytes showed that 2,3-dehydrosilybin can be metabolized by conjugation (phase II) reactions, namely glucuronidation or sulfation, without prior phase I oxidations [16]. Human hepatocytes are a complex metabolic system [18], and therefore it can be assumed that glucuronidation and sulfation may be the relevant biotransformation pathways of 2,3-dehydrosilybin in the human organism. In addition, UDP-glucuronosyltransferases (UGTs) and sulfotransferases (SULTs) are expected to be involved in the metabolism of 2,3-dehydrosilybin, but the specific isozymes involved are not known. Since the existing metabolism studies were performed only with a mixture of 2,3-dehydrosilybin enantiomers, the aim of this study was to investigate the biotransformation of pure 2,3-dehydrosilybin A and 2,3-dehydrosilybin B in human hepatocytes and to identify human UGT and SULT enzymes with activity toward individual 2,3-dehydrosilybin enantiomers.

## 2. Materials and Methods

### 2.1. Tested Compounds

2,3-Dehydrosilybin A (98.4% purity) and 2,3-dehydrosilybin B (93.2% purity) were prepared from silybin A and silybin B, respectively, by oxidation with iodine in glacial acetic acid with sodium acetate as described previously [4,19]. The purity of 2,3-dehydrosilybin enantiomers was determined by high-performance liquid chromatography as described in [3]. For the isolation and separation of silybin A and B from commercially available silymarin (Liaoning Senrong Pharmaceutical, Panjin, China), see Refs. [20,21]. For biotransformation experiments, fresh 50 mM stock solutions of 2,3-dehydrosilybin A and B in dimethyl sulfoxide (DMSO; Sigma-Aldrich, St. Louis, MO, USA) were used.

### 2.2. Incubation of 2,3-Dehydrosilybin A and B with Human Hepatocytes

The use of human hepatocytes was approved by the Ethics Committee of University Hospital Olomouc, Czech Republic (approval No. 119/07). The samples of human liver were obtained from multiorgan donors. Hepatocytes were isolated using two-step collagenase perfusion, and resuspended in serum-free medium containing Williams’ medium E, Ham’s F-12 medium, and additives as described previously [22]. Hepatocyte cultures used in the study were prepared from liver samples of three donors: a 45-year-old man (culture LH81), a 56-year-old man (culture LH83), and a 47-year-old woman (culture LH84).

Suspensions of human hepatocytes (4 × 10^6^ cell/mL) were incubated with 0.1% (*v*/*v*) DMSO (control) or with 50 µM 2,3-dehydrosilybin A or B (in 0.1% (*v*/*v*) DMSO). After 1 h of incubation at 37 °C and 160 rpm in an ES-20 Environmental Shaker (Biosan, Riga, Latvia), the cells and media were separated by centrifugation for 5 min at 150× *g* and 4 °C, and stored at −80 °C until their analysis by ultra-high-performance liquid chromatography coupled with tandem mass spectrometry (UHPLC-MS).

### 2.3. Incubation of 2,3-Dehydrosilybin A and B with Human UGTs

Glucuronidation of the tested compounds by individual UGT enzymes was examined using Corning Supersomes (Discovery Labware, Woburn, MA, USA), i.e., microsomes from baculovirus-transfected insect cells expressing the recombinant human UGTs 1A1, 1A3, 1A4, 1A6, 1A7, 1A8, 1A9, 1A10, 2B4, 2B7, 2B10, 2B15, or 2B17. The incubations were performed in 0.25 mL of Tris–HCl buffer (pH 7.4; 100 mM) containing 8 mM MgCl_2_, 25 µg/mL alamethicin, 50 µM 2,3-dehydrosilybin A or B, 0.2 mg/mL microsomal protein and 2 mM UDP-glucuronic acid (Sigma-Aldrich, St. Louis, MO, USA), with a final concentration of DMSO of 0.6% (*v*/*v*). Control samples were prepared by incubating the tested compounds with Corning Supersomes insect cell control microsomes (Discovery Labware, Woburn, MA, USA), which lack UGT activity. All samples were incubated for 30 min at 37 °C and 300 rpm in a Thermomixer Comfort (Eppendorf, Hamburg, Germany), and then stored at −80 °C until their analysis by UHPLC-MS.

### 2.4. Incubation of 2,3-Dehydrosilybin A and B with Human Sulfotransferases

Sulfation of the tested compounds by individual sulfotransferases was examined using cytosolic fractions from *Escherichia coli* expressing recombinant human SULTs 1A1*1, 1A1*2, 1A2, 1A3, 1B1, 1C2, 1C4, 1E1, or 2A1 (Cypex, Dundee, UK). The incubations were performed in 0.2 mL of potassium phosphate–HCl buffer (pH 7.4; 50 mM) containing 5 mM MgCl_2_, 10 mM dithiothreitol, 50 µM 2,3-dehydrosilybin A or B (in 0.1% (*v*/*v*) DMSO), 50 µg/mL *E. coli* cytosol protein, and 120 µM 3′-phosphoadenosine 5′-phosphosulfate (PAPS; Sigma-Aldrich, St. Louis, MO, USA). Control samples were prepared by incubating the tested compounds in the absence of PAPS and/or the cytosolic fraction. All samples were incubated for 30 min at 37 °C and 300 rpm in a Thermomixer Comfort (Eppendorf, Hamburg, Germany), and then stored at –80 °C until their analysis by UHPLC-MS.

### 2.5. UHPLC-MS^E^ Analysis

For the analysis of biotransformation products, hepatocytes collected by centrifugation were resuspended in 0.4 mL of methanol containing 5% (*v*/*v*) acetic acid, and disintegrated by sonication on ice (10 cycles, 0.5 s pulses, 50% amplitude) using a UP200s Ultrasonic Processor equipped with a Sonotrode S2 sonicator probe (Hielscher, Teltow, Germany). The samples of media and incubation mixtures with UGT and SULT enzymes were thawed and mixed 1:1 (*v*/*v*) with methanol containing 5% (*v*/*v*) acetic acid. All samples were centrifuged for 5 min at 12,000× *g* and 4 °C, and the supernatants were analyzed. The analyses were performed using an Acquity UPLC I-Class system (Waters, Milford, MA, USA) including a solvent manager, sample manager, and column manager with a Kinetex 2.6 µm Polar C18 column (100 × 2.1 mm i.d.; Phenomenex, Torrance, CA, USA). The mobile phase A was an aqueous solution of 5 mM ammonium acetate, pH 3, and the mobile phase B was methanol. The column oven was maintained at 35 °C, and the elution was performed at a flow rate of 0.5 mL/min with the linear gradient: 0–2 min 10% B, 2–8 min 10–55% B, 8–8.5 min 55–70% B, 8.5–9 min 70–10% B, followed by equilibration at 10% B. The samples were maintained in the autosampler at 10 °C, and the injection volume was 2 µL. The electrospray ionization source of a Synapt G2-S Mass Spectrometer (Waters, Milford, MA, USA) was operated in negative mode with the following settings: capillary voltage 2.25 kV, sampling cone 35 V, source temperature 120 °C, desolvation temperature 300 °C, cone gas flow 25 L/h, and desolvation gas flow 600 L/h. Data in the range of 50–1200 Da were collected with a scan time of 0.2 s, and a sodium formate solution in acetonitrile was used for the instrument calibration. Data were automatically processed and corrected for mass error during acquisition with leucine enkephalin as an external reference. Data were obtained using two interleaved scan functions (MS^E^ experiments), enabling the simultaneous acquisition of both low-collision-energy (4 eV) and high-collision-energy (15–30 eV) mass spectra. Post-acquisition analysis of the data was performed using the software Metabolynx v4.1 (Waters, Milford, MA, USA). MS data with ppm ≥ 5 were not considered.

The validation of the UHPLC-MS method with 2,3-dehydrosilybin A and B included tests of selectivity, linearity, precision, and accuracy. The selectivity was evaluated by comparing the control samples with samples spiked with the tested compounds. The linearity was tested over the concentration range of 2.5–50 µM, and the values of the coefficient of determination (R^2^) were found to be 0.9981–0.9988. The inter-day precision and accuracy, tested in three replicates, were expressed as a relative standard deviation (RSD) and relative error (RE), respectively. The values of RSD did not exceed 15%. The values of RE did not exceed 10%, with the exception of 2,3-dehydrosilybin B at a concentration of 5 µM (13.2%).

## 3. Results and Discussion

### 3.1. Biotransformation of 2,3-Dehydrosilybin A and B in Human Hepatocytes

2,3-Dehydrosilybin is a better radical scavenger and stronger antioxidant than its natural precursor silybin due to the presence of a 2,3-double bond [4,5]. Therefore, it is attracting increasing attention, especially in medicinal chemistry and experimental pharmacology. In this study, we investigated the biotransformation of pure 2,3-dehydrosilybin enantiomers in human hepatocytes and identified the major phase II enzymes that can catalyze their conjugation. Using UHPLC-MS analyses, we found that 2,3-dehydrosilybin A and B were metabolized to a similar extent by human hepatocytes at a non-cytotoxic concentration of 50 µM [16]. Thus, the proportions of the parent compounds in the cells after 1 h of incubation were 85% and 83%, respectively. Moreover, five types of metabolites were detected for each enantiomer, and the biotransformations included both phase I and phase II reactions (Table 1).

**Figure 2:**
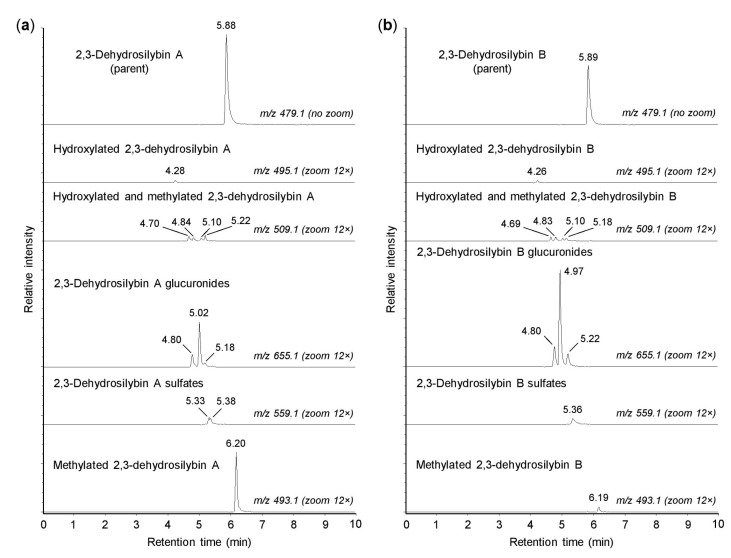
Biotransformation of 2,3-dehydrosilybin A and B by human hepatocytes. Human hepatocytes were incubated for 1 h with 50 µM 2,3-dehydrosilybin A (panel **a**) or 2,3-dehydrosilybn B (panel **b**), collected by centrifugation and analyzed by ultra-high-performance liquid chromatography coupled with tandem mass spectrometry (UHPLC-MS). The signal intensity in chromatograms was adjusted to the same value before using the zoom tool.

As shown in Figure 2, both 2,3-dehydrosilybin A and 2,3-dehydrosilybin B (*m*/*z* 479.0974) were transformed into one monohydroxylated metabolite (*m*/*z* 495.0921) and four metabolites generated by hydroxylation and methylation (*m*/*z* 509.1084). The formation of hydroxylated products confirmed that cytochromes P450 might play a role in the biotransformation of 2,3-dehydrosilybin enantiomers [16]. Moreover, the parent molecules of 2,3-dehydrosilybin A and B bear five hydroxyl groups, so they were also metabolized by conjugation reactions without prior oxidative modifications. We found that 2,3-dehydrosilybin A and B were each converted to three monoglucuronides (*m*/*z* 655.1287), one or two monosulfates (*m*/*z* 559.0513), and one monomethyl derivative (*m*/*z* 493.1132) (Figure 2). The monoglucuronides were the most abundant metabolites overall, while the other metabolite species were formed only to a lesser extent (Table 1). The retention times of all biotransformation products, except the methyl derivatives, were lower than the retention times of the parent compounds (Figure 2).

The results demonstrated that both 2,3-dehydrosilybin enantiomers were metabolized by human hepatocytes in a similar pattern, where the products of the direct conjugations predominated over those derived by oxidative biotransformation. It is also worth noting that the main difference between the biotransformation of 2,3-dehydrosilybin enantiomers was found in their methylation, where 2,3-dehydrosilybin A was more susceptible to this type of conjugation. This study further focused on the conjugation of 2,3-dehydrosilybin enantiomers with glucuronic acid and sulfate. These conjugation reactions are known to play an important role in phase II xenobiotic biotransformation, and in the case of flavonoid-type compounds, they are associated with increased water solubility, biological inactivation, and elimination of target molecules from the body [23,24].

### 3.2. Glucuronidation of 2,3-Dehydrosilybin A and B by Individual Human UGTs

**Figure 3:**
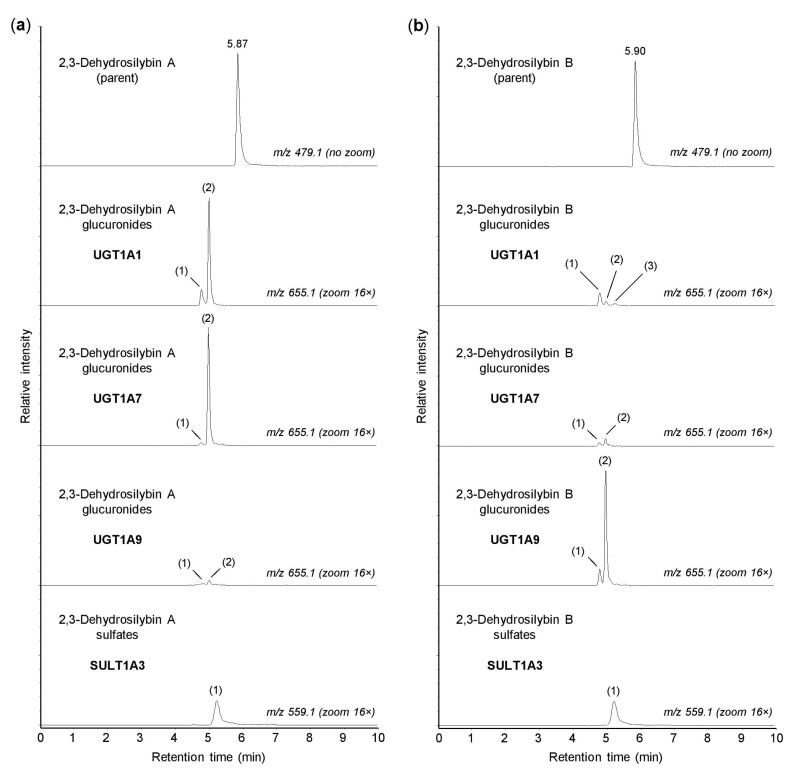
Glucuronidation and sulfation of 2,3-dehydrosilybin A and B by recombinant human enzymes. Human UGT1A1, UGT1A7, UGT1A9, and SULT1A3 were incubated for 30 min with 50 µM 2,3-dehydrosilybin A (panel **a**) or 2,3-dehydrosilybin B (panel **b**), and the biotransformation products were analyzed by UHPLC-MS. The peaks of glucuronides and sulfates are marked with numbers (in brackets) corresponding to those in Table 2 and Table 3. The signal intensity in chromatograms was adjusted to the same value before using the zoom tool.

In the human organism, the conjugation reactions with glucuronic acid are catalyzed by a family of 22 UDP-glucuronosyltransferases, localized in the membranes of the smooth endoplasmic reticulum [25]. In this study, the glucuronidation of 2,3-dehydrosilybin A and B was investigated using 13 recombinant human enzymes from the UGT1A and UGT2B subfamilies, which are known to be involved in the biotransformation of xenobiotics [25]. We found that UGTs 1A1, 1A3, 1A7, 1A8, 1A9, and 1A10 were able to catalyze the glucuronidation of both 2,3-dehydrosilybin A and 2,3-dehydrosilybin B, whereas UGTs 1A4, 1A6, 2B4, 2B7, 2B10, 2B15, and 2B17 were inactive (Table 2). In addition, up to three glucuronidation products were detected for each enantiomer, and all of these potential metabolites were monoglucuronides (*m*/*z* 655.1287). Of the active enzymes, UGTs 1A3, 1A8, and 1A10 showed low activity toward both 2,3-dehydrosilybin enantiomers, with the proportion of glucuronides not exceeding 1% after 30 min of incubation. In contrast, UGTs 1A1, 1A7, and 1A9 showed higher activity but also higher stereoselectivity, in which the proportions of glucuronides reached about 7% for one enantiomer and remained below 1% for the other (Table 2). As shown in Figure 3, UGTs 1A1 and 1A7 showed higher activity with 2,3-dehydrosilybin A, while UGT1A9 was more active with 2,3-dehydrosilybin B. It should be mentioned that all six UGT enzymes producing 2,3-dehydrosilybin glucuronides are expressed in the small intestine, and UGTs 1A1, 1A3, and 1A9 are also present in the liver [25,26].

### 3.3. Sulfation of 2,3-Dehydrosilybin A and B by Individual Human Sulfotransferases

The sulfation of xenobiotics, as well as small endogenous molecules, is catalyzed by the cytosolic sulfotransferases. In humans, this enzyme family comprises 13 members that are differentially expressed in many tissues [27]. In this study, we investigated the sulfation of 2,3-dehydrosilybin A and B using recombinant human SULTs 1A1*1, 1A1*2, 1A2, 1A3, 1B1, 1C2, 1C4, 1E1, and 2A1. We found that all enzymes tested, with the exception of SULT2A1, were able to sulfate both 2,3-dehydrosilybin A and 2,3-dehydrosilybin B (Table 3). Each enantiomer was converted to one or two monosulfates (*m*/*z* 559.0513) by the active enzymes, but the amounts of sulfation products were small. After 30 min of incubation, the highest amount of sulfates (approximately 2%) was found with SULT1A3 (Figure 3), and at least 1% sulfation was also found with SULTs 1B1, 1C4, and 1E1 (Table 3). Among the active enzymes, SULTs 1A1, 1A3, 1B1, and 1E1 are the major sulfotransferases in the small intestine, and the same enzymes, except SULT1A3, are also expressed in the liver [24,28]. On the other hand, SULTs 1C2 and 1C4 are mainly present in human tissues during fetal development, and SULT1A2 is not expressed at the protein level [27].

## 4. Conclusions

We have demonstrated, for the first time, that both 2,3-dehydrosilybin A and 2,3-dehydrosilybin B may undergo four types of metabolic reactions in isolated human hepatocytes, namely, hydroxylation, methylation, sulfation, and glucuronidation. In hepatocytes, both 2,3-dehydrosilybin enantiomers were preferentially metabolized to glucuronides, while the sulfates, methyl derivatives, hydroxyl derivatives, and methylated hydroxyl derivatives were produced to a lesser extent. We have also demonstrated that the glucuronidation and sulfation of 2,3-dehydrosilybin A and B can be catalyzed by UGTs 1A1, 1A3, 1A7, 1A8, 1A9, and 1A10, and SULTs 1A1*1, 1A1*2, 1A2, 1A3, 1B1, 1C2, 1C4, and 1E1, respectively. Considering the tissue distribution of UGT and SULT enzymes, we conclude that multiple human enzymes may be involved in the glucuronidation and sulfation of 2,3-dehydrosilybin A and B, and these conjugations may be expected to occur both in the intestine and in the liver. Since 2,3-dehydrosilybin enantiomers were methylated in human hepatocytes either directly or after hydroxylation, we suggest that further research could investigate whether catechol *O*-methyltransferase is involved in the metabolism.

## Figures and Tables

**Table 1:** Biotransformation of 2,3-dehydrosilybin A and 2,3-dehydrosilybin B in human hepatocytes.

			Semiquantitative Percentage ^2^			
			2,3-Dehydrosilybin A		2,3-Dehydrosilybin B	
Metabolic Reaction	Formula	*m*/*z* ^1^	Cells	Medium	Cells	Medium
Parent compound	C_25_H_20_O_10_	479.0974	85.1	70.5	83.1	75.8
Hydroxylation	C_25_H_20_O_11_	495.0921	0.4	0.0	0.4	0.0
Hydroxylation and methylation	C_26_H_22_O_11_	509.1084	0.9	0.7	1.0	1.1
Glucuronidation	C_31_H_28_O_16_	655.1287	9.2	24.7	13.0	20.2
Sulfation	C_25_H_20_O_13_S	559.0513	2.0	2.8	2.2	2.9
Methylation	C_26_H_22_O_10_	493.1132	2.4	1.3	0.3	0.0

Human hepatocytes were incubated for 1 h with 50 µM 2,3-dehydrosilybin A or B, and then cells and culture media were separately analyzed by UHPLC-MS. ^1^ The *m*/*z* values for pseudomolecular ions [M − H]^−^. ^2^ The semiquantitative percentage values were evaluated based on the peak area values. The sum of a given parent compound plus all of its biotransformation products gives 100%. The values are means from three hepatocyte cultures.

**Table 2:** Glucuronidation of 2,3-dehydrosilybin A and 2,3-dehydrosilybin B by human UDP-glucuronosyltransferases.

		Semiquantitative Percentage ^2^												
		UGT												
Compound/Metabolite	t_R_ ^1^ (min)	1A1	1A3	1A4	1A6	1A7	1A8	1A9	1A10	2B4	2B7	2B10	2B15	2B17
2,3-Dehydrosilybin A	5.87	93.3	99.9	100.0	100.0	93.0	99.5	99.7	99.9	100.0	100.0	100.0	100.0	100.0
Glucuronide (1)	4.80	1.1	0.1	–	–	0.2	0.3	0.1	<0.1	–	–	–	–	–
Glucuronide (2)	5.02	5.6	–	–	–	6.8	0.2	0.2	<0.1	–	–	–	–	–
Glucuronide (3)	5.43	–	–	–	–	<0.1	–	–	–	–	–	–	–	–
2,3-Dehydrosilybin B	5.90	99.1	99.9	100.0	100.0	99.5	99.6	92.9	99.8	100.0	100.0	100.0	100.0	100.0
Glucuronide (1)	4.80	0.6	<0.1	–	–	0.2	0.1	0.9	<0.1	–	–	–	–	–
Glucuronide (2)	5.02	0.2	<0.1	–	–	0.3	0.3	6.2	0.1	–	–	–	–	–
Glucuronide (3)	5.24	0.1	–	–	–	–	–	–	0.1	–	–	–	–	–

Recombinant human UGTs 1A1, 1A3, 1A4, 1A6, 1A7, 1A8, 1A9, 1A10, 2B4, 2B7, 2B10, 2B15, and 2B17 were incubated for 30 min with 50 µM 2,3-dehydrosilybin A or B. Parent compounds (*m*/*z* 479.0974) and their monoglucuronides (*m*/*z* 655.1287) were then analyzed by UHPLC-MS. ^1^ Retention time. ^2^ The semiquantitative percentage values were evaluated based on the peak area values. The sum of a given parent compound plus its glucuronidation products gives 100%. The values are means from three experiments. The minus sign (–) indicates that a given metabolite was not detected.

**Table 3:** Sulfation of 2,3-dehydrosilybin A and 2,3-dehydrosilybin B by human sulfotransferases.

		Semiquantitative Percentage ^2^								
		SULT								
Compound/Metabolite	t_R_ ^1^ (min)	1A1*1	1A1*2	1A2	1A3	1B1	1C2	1C4	1E1	2A1
2,3-Dehydrosilybin A	5.89	99.8	99.7	99.2	97.8	98.6	99.8	98.7	99.0	100.0
Sulfate (1)	5.25	<0.1	<0.1	0.1	2.2	1.3	0.2	0.8	0.1	–
Sulfate (2)	5.50	0.2	0.2	0.7	–	0.1	–	0.5	0.9	–
2,3-Dehydrosilybin B	5.92	99.6	99.6	99.6	98.0	99.2	99.7	98.1	98.5	100.0
Sulfate (1)	5.26	0.1	<0.1	<0.1	2.0	0.2	0.3	1.9	–	–
Sulfate (2)	5.45	0.3	0.4	0.4	–	0.6	–	–	1.5	–

Recombinant human SULTs 1A1*1, 1A1*2, 1A2, 1A3, 1B1, 1C2, 1C4, 1E1, and 2A1 were incubated for 30 min with 50 µM 2,3-dehydrosilybin A or B. Parent compounds (*m*/*z* 479.0974) and their monosulfates (*m*/*z* 559.0513) were then analyzed by UHPLC-MS. ^1^ Retention time. ^2^ The semiquantitative percentage values were evaluated based on the peak area values. The sum of a given parent compound plus its sulfation products gives 100%. The values are means from three experiments. The minus sign (–) indicates that a given metabolite was not detected.

## Data Availability

The data presented in this study are available in this article.
